# LEGUME-MAIZE ROTATION OR RELAY? OPTIONS FOR ECOLOGICAL INTENSIFICATION OF SMALLHOLDER FARMS IN THE GUINEA SAVANNA OF NORTHERN GHANA

**DOI:** 10.1017/S0014479718000273

**Published:** 2018-07-10

**Authors:** M. KERMAH, A. C. FRANKE, B. D. K. AHIABOR, S. ADJEI-NSIAH, R. C. ABAIDOO, K. E. GILLER

**Affiliations:** † Plant Production Systems, Wageningen University, P.O. Box 430, 6700 AK Wageningen, the Netherlands; § Soil, Crop and Climate Sciences, University of the Free State, P.O. Box 339, Bloemfontein 9300, South Africa; §§ CSIR-Savanna Agricultural Research Institute, P.O. Box 52, Tamale, Ghana; †† International Institute of Tropical Agriculture, P.O. Box TL 06, Tamale, Ghana; ¶ Department of Theoretical and Applied Biology, Kwame Nkrumah University of Science and Technology, PMB, Kumasi, Ghana

## Abstract

Soil nutrient constraints coupled with erratic rainfall have led to poor crop yields and occasionally to crop failure in sole cropping in the Guinea savanna of West Africa. We explored different maize-grain legume diversification and intensification options that can contribute to mitigating risks of crop failure, increase crop productivity under different soil fertility levels, while improving soil fertility due to biological N_2_-fixation by the legume. There were four relay patterns with cowpea sown first and maize sown at least 2 weeks after sowing (WAS) cowpea; two relay patterns with maize sown first and cowpea sown at least 3 WAS maize in different spatial arrangements. These were compared with groundnut-maize, soybean-maize, fallow-maize and continuous maize rotations in fields high, medium and poor in fertility at a site each in the southern (SGS) and northern (NGS) Guinea savanna of northern Ghana. Legumes grown in the poorly fertile fields relied more on N_2_-fixation for growth leading to generally larger net N inputs to the soil. Crop yields declined with decreasing soil fertility and were larger in the SGS than in the NGS due to more favourable rainfall and soil fertility. Spatial arrangements of relay intercrops did not have any significant impact on maize and legume grain yields. Sowing maize first followed by a cowpea relay resulted in 0.18–0.26 t ha−1 reduction in cowpea grain yield relative to cowpea sown from the onset. Relaying maize into cowpea led to a 0.29–0.64 t ha−1 reduction in maize grain yield relative to maize sown from the onset in the SGS. In the NGS, a decline of 0.66 and 0.82 t ha−1 in maize grain yield relative to maize sown from the onset was observed due to less rainfall received by the relay maize. Groundnut and soybean induced 0.38–1.01 t ha−1 more grain yield of a subsequent maize relative to continuous maize, and 1.17–1.71 t ha−1 more yield relative to relay maize across both sites. Accumulated crop yields over both years suggest that sowing maize first followed by cowpea relay is a promising ecological intensification option besides the more common legume–maize rotation in the Guinea savanna, as it was comparable with soybean–maize rotation and more productive than the other treatments.

## INTRODUCTION

The increasing population in sub-Saharan Africa, and its associated growing demand for food require the intensification of crop production (Vanlauwe et al., 2014). In the Guinea savanna agro-ecological zone of West Africa, continuous cereal-based cropping without adequate soil nutrient replacement has resulted in a decline in soil fertility with corresponding poor crop yields (Bationo et al., 1998). The duration of fallow period to regenerate soil fertility has become increasingly shorter as a result of population pressure and the competing demands for arable land (Agyare et al., 2006; Sauerborn *et al*., 2000). Crop or cropping system diversification with the integration of grain legumes is needed, as grain legumes can biologically fix atmospheric N2 to improve soil fertility and increase (and/or sustain) crop yields. Grain legumes also provide protein-rich edible seeds that contribute substantially to household food, nutrition and income security (Giller, 2001).

Legume-cereal rotations have gained prominence in the Guinea savanna region of West Africa, and increased yields of maize (*Zea mays* L.) succeeding legumes relative to continuous sole maize cropping are well documented (e.g. Agyare *et al*., 2006; Franke *et al*., 2004, 2008; Oikeh *et al*., 1998; Sanginga *et al*., 2002; Sauerborn *et al*., 2000; Yusuf *et al*., 2009a, 2009b). The increased yields of maize following legumes are partly due to N contributed by the legumes through biological N2 fixation to improve soil fertility (Giller, 2001; Yusuf *et al*., 2009a). Non-N benefits, such as reduced pests and diseases incidence, increased soil microbial biomass and activity and improved soil properties may also contribute to the increased yield of maize in rotation with legumes (Franke *et al*., 2018; Giller, 2001; Yusuf *et al*., 2009b).

Soil fertility differs markedly across farmers’ fields in the Guinea savanna zone of West Africa, and may strongly influence the benefits of legume-maize rotations (Oikeh *et al*., 1998). For instance, residual biomass production as well as the proportion of N derived from N_2_-fixation (%Ndfa) by grain legumes, N_2_ fixed, N uptake and net N contribution to the soil differ depending on the fertility of the soil (Kermah *et al*., 2018). Further, erratic rainfall pattern presents a risk for farmers considering that the Guinea savanna has only one cropping season a year and a bad harvest can seriously threaten food, nutrition and income security (Sauerborn *et al*., 2000). Legume-maize diversification and intensification systems that maintain the recommended planting density of sole maize (the main crop in northern Ghana) with a grain legume added may provide yield in the event of poor rainfall and failure of the maize (Rusinamhodi *et al*., 2012). To date, the focus of many studies has been on sole crop legume-maize rotations and distinct alternate arrangement of intercrops. These trials have been conducted mainly on experimental stations (e.g. Agyare *et al*., 2006; Sanginga *et al*., 2002; Sauerborn *et al*., 2000; Yusuf *et al*., 2009a). In addition, soil fertility status was rarely taken into account in evaluating legume-maize rotations and intercrop systems in the Guinea savanna region of West Africa. Greater productivity of maize and grain legume intercrops when sown within the same row compared with sole crops, or distinct 1 to 1 and 2 to 2 alternate row arrangement of intercrops has been reported in the Guinea savanna of northern Ghana (Kermah *et al*., 2017), southern Mali (Falconnier *et al*., 2016) and central Mozambique (Rusinamhodzi *et al*., 2012).

We therefore studied the agronomic performance of short duration maize and cowpea (*Vigna unguiculata* (L.) Walp) relay cropping patterns sown within the same row. These were compared with continuous maize, soybean (*Glycine max* (L.) Merr.)-maize, groundnut (*Arachis hypogaea* L.)-maize and natural fallow-maize rotations under different soil fertility levels in farmers’ fields in the southern and northern Guinea savanna agro-ecological zones of northern Ghana. Our aim was to assess and explore a variety of legume-maize diversification and intensification options that could be offered to smallholder farmers to increase crop productivity.

## MATERIALS AND METHODS

### Study sites

On-farm trials were carried out in the 2013 (year 1) and 2014 (year 2) cropping seasons at Kpataribogu (9°58' N, 0°40' W) in the Karaga District (southern Guinea savanna, SGS) and at Bundunia (10°51' N, 1°04' W) in the Kassena-Nankana East Municipal (northern Guinea savanna, NGS). The unimodal rainfall regime at both sites gives rise to a single cropping season that starts with the onset of rainfall from May/June–October in the SGS and June/July–October in the NGS with an erratic distribution pattern. A delay in the onset of rainfall has been observed in recent seasons at both sites. Soils at both sites are generally sandy and poor in fertility. In the Interim Ghana Soil Classification System (Adjei-Gyapong and Asiamah, 2002), the soils are classified as Savanna Ochrosols and Groundwater Laterites.

### Experimental design and trial management

Prior to the start of the trials in year 1, three fields each representing highly fertile (HF), medium fertile (MF) and poorly fertile (LF) fields were selected at each site based on farmers’ knowledge and support from Agricultural Extension Officers. Past crop performance and/or yield were the main criteria used by the farmers to classify the fields. The fields initially classified as HF and MF fields in the SGS were swopped after soil characteristics became available. Each field was previously under monocropped maize, legume or cotton in the three years preceding the start of the trials. Before land preparation in year 1, twelve cores of soil samples were taken at 0–15 cm depth per field, bulked and mixed thoroughly to obtain a composite soil per field. Sub-samples of about 1 kg soil per field were analysed for pH (1:2.5 soil:water suspension), organic C by the Walkley and Black wet oxidation method (Nelson and Sommers, 1982), total N by the Kjeldahl digestion method (Tel and Hagatey, 1984), available P by Olsen method (Olsen *et al*., 1954), exchangeable K, Mg and Ca in 1.0 M ammonium acetate extracts (Nelson and Sommers, 1982) and texture (hydrometer method).

**Figure 1:**
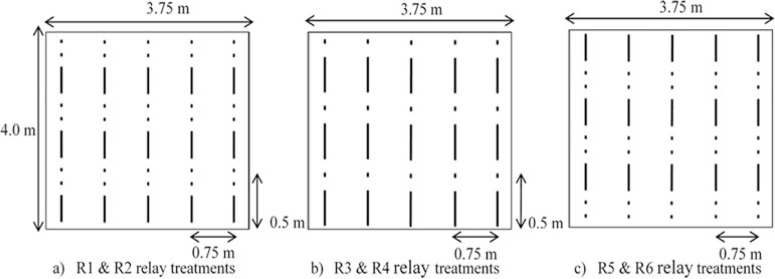
A graphical representation of the relay planting arrangements: (a) R1 and R2 relay treatments with two stands of cowpea alternated with a stand of maize with the maize sown later, (b) R3 and R4 relay treatments with a stand each of cowpea and maize alternated with maize sown later, and (c) R5 and R6 treatments where a maize stand was alternated with two cowpea stands with the cowpea sown later. A hyphen (–) represents a maize stand and a dot (.) for a cowpea stand, and not drawn to scale.

The trial consisted of 10 treatments: four cowpea-maize relay treatments (treatments R1–R4) and two maize–cowpea relay treatments (treatments R5 and R6), a groundnut–maize (GN–MZ), a soybean–maize (SB–MZ) and a natural fallow– maize (FL–MZ) rotations and a continuous sole maize system (MZ–MZ). Details of the treatments are presented in Table 1. In relay treatments R1, R2, R5 and R6, maize was spaced at 50 cm within a row with two maize plants per stand and alternated with two equally spaced cowpea stands. In relay treatments R3 and R4, maize was spaced at 25 cm (one maize plant per stand) and alternated with one cowpea stand within a row. Figure 1 provides a graphical representation of the relay planting arrangements. Intra-row spacing for sole maize was 25 cm. Inter-row spacing was 75 cm for all treatments. Cowpea and maize were sown at a density of 53,333 plants ha−1 in all applicable treatments; groundnut and soybean (spaced 10 cm intra row) had a density of 133,333 and 400,000 plants ha−1 , respectively. The trial was set-up in a randomised complete block design replicated four times per fertility level at each site.

**Table 1:** Description of treatments evaluated in experiment.

	Year 1				Year 2			
Treatment	Crop	Variety^a^	Sowing time^c^	Plants/ stand	Crop	Variety	Sowing time^c^	Plants/ stand
CP–MZ relay	Cowpea	Songotra	0	1	Cowpea	Songotra	0	1
2:1 stands (R1)	Maize	Dorke SR	3	2	Maize	Dorke SR	2	2
CP–MZ relay	Cowpea	Songotra	0	1	Cowpea	Songotra	0	1
2:1 stands (R2)	Maize	Dorke SR	6	2	Maize	Dorke SR	4	2
CP–MZ relay	Cowpea	Songotra	0	1	Cowpea	Songotra	0	1
1:1 stands (R3)	Maize	Dorke SR	3	1	Maize	Dorke SR	2	1
CP–MZ relay	Cowpea	Songotra	0	1	Cowpea	Songotra	0	1
1:1 stands (R4)	Maize	Dorke SR	6	1	Maize	Dorke SR	4	1
MZ–CP relay	Maize	Dorke SR	0	2	Maize	Dorke SR	0	2
1:2 stands (R5)	Cowpea	Bawutawuta	6	1	Cowpea	Songotra	3	1
MZ–CP relay	Maize	Dorke SR	0	2	Maize	Dorke SR	0	2
1:2 stands (R6)	Cowpea	Bawutawuta	9	1	Cowpea	Songotra	5	1
GN–MZ rotation	Groundnut	Samnut 22/Chinese^b^	0	1	Maize	Obatanpa	0	1
SB–MZ rotation	Soybean	Jenguma	0	3	Maize	Obatanpa	0	1
MZ–MZ rotation	Maize	Obatanpa	0	1	Maize	Obatanpa	0	1
FL–MZ rotation	–	–	–	–	Maize	Obatanpa	0	1

CP = cowpea; SB = soybean; GN = groundnut; MZ = maize; FL = fallow.

a In relay treatments R1–R4, maize variety Dodzi was used in the northern Guinea savanna (NGS).

b Samnut 22 used in the southern Guinea savanna (SGS) and ‘Chinese’ variety used in the NGS.

v Weeks after sowing the first crop.

Land preparation followed common practices of farmers at each site: ploughing by tractor followed by manual ridging in the SGS and ploughing and ridging by tractor in the NGS. Seeds of all crops were sown on the top of the ridges at both sites. The first crop in each treatment was sown simultaneously on June 30–July 1 in the SGS and July 16–17 in the NGS in year 1 (the onset of rain in the NGS was late), and on July 17 in the SGS and July 15 in the NGS in year 2. In year 1, cowpea harvest coincided with the peak of rainfall in August and September, resulting in poor drying of pods and discoloured grains that were largely rejected by farmers. To avoid this, cowpea sowing was done relatively late in the SGS in year 2. The relay crops in R1 to R6 were sown into the first crops at different times specified in Table 1. The sowing times of the relay crops were altered in year 2 due to the failure of some relay crops in year 1 partly due to the late sowing of those crops.

In year 1, urea at a rate of 50 kg N ha^−1^ was applied uniformly to each maize treatment in two equal doses at 3 and 6 weeks after sowing (WAS) of maize. In relay plots, urea was applied only to maize plants. In year 2, no N fertiliser was applied to maize in any treatment in order to measure the residual N and non-N effects of the grain legumes on the succeeding maize. TSP at a rate of 25 kg P ha^−1^ (57 kg P_2_ O_5_ ha^−1^ ) and muriate of potash at 30 kg K ^ha−1^ (36 kg K_2_ O ^ha−1^ ) were applied uniformly to all treatments at sowing in both years. All fertilisers were placed at 3 cm depth, 5 cm away from the plants and covered. Weeding was done with a hoe in each treatment at 3 and 6 WAS. A third weeding was done in R1–R6 at 9 WAS the first crop. Seeds of soybean were inoculated with a commercial inoculant, Legumefix (LegumeTechnology, UK) containing Bradyrhizobium japonicum strain 532c (re-isolated in Brazil from strain USDA 442 Wisconsin, USA) at a rate of 5 g of inoculant per kg of seeds at sowing. Cowpea in all treatments was sprayed twice with lambda-cyhalothrin (SGS) and cypadem 43.6 EC (36 g cypamethrin and 400 g dimethoate per litre) (NGS) in the form of an emulsifiable concentrate at 0.75–1.00 L ha−1 per insecticide at flowering and podding stages, contingent on pests incidence and pressure [i.e. flower thrips (e.g. *Megalurothrips sjostedti* Tryb.) and pod borers (e.g. *Maruca vitrata* Fab.)] in accordance with farmers’ practice.

### Rainfall, crop yield, N_2_-fixation and N balance measurements

Daily rainfall during the growing season at each site was measured using rain gauges. Above-ground legume biomass was sampled in a 1.0 m × 2.25 m area at mid-pod filling stage and separated into shoots and pods. Total and sub-sample fresh weights of shoots and pods were taken in the field. At maturity, a 2.0 × 2.25 m area was harvested for legume and maize yield determination. Total and sub-sample fresh weights of legume pods, maize cobs and stover of all crops were recorded in the field. Fresh weight to dry weight conversion factors for the different plant parts were used to calculate the dry weights of the sub-samples. These conversion factors were derived from experimental data of trials conducted in the Guinea savanna of northern Ghana and Nigeria and previously reported by Kermah *et al*. (2017, 2018). Above-ground legume biomass and stover yield of all crops are presented on a dry weight basis, maize grain at 14% and legume grain at 12% moisture contents. N uptake by legumes in year 1 was estimated with N concentration values taken from Nijhof (1987). These were cowpea grain: 2.90%, stover: 1.73%; soybean grain: 6.10%, stover: 1.05% and groundnut grain: 4.50%, stover: 1.40%. Maize N uptake in both years was calculated with maize grain and stover N concentrations measured in year 2.

A selection of non-legume broad-leaved reference weeds were sampled from the fallow plots in each block at the biomass sampling stage of the legumes. The mean δ15 N enrichment of these reference weeds was used for estimating the proportion of N derived from the atmosphere (%Ndfa) by the legumes using the ^15^ N natural abundance method (Unkovich *et al*., 2008). N_2_-fixation was measured for the HF and LF fields only. The separately measured δ^15^ N of the legume shoots and pods harvested at mid-pod filling was used to calculate the weighted δ^15^ N for the whole shoots:

$$\text{=}\left[\left(\text{shoot}{\text{N×δ}}^{\text{15}}\text{N shoot}\right)\text{+}\left(\text{pod}{\text{N×δ}}^{\text{15}}\text{N pod}\right)\right]\text{/}\left(\text{shoot N+pod N}\right)\text{.}\left(\text{1}\right)$$( 1 )

Shoot N was calculated as: %N shoot/100 × shoot dry matter yield (kg ha^−1^ ), while pod N was determined as: %N pod/100 × pod dry matter yield (kg ha^−1^ ). %Ndfa was estimated using the weighted δ^15^ N of the whole shoot following Unkovich *et al*. (2008) as

$$\%\text{Ndfa=}\left[\left(\delta^{15}{\text{N}}_{\text{ref}}-\delta^{15}{\text{N}}_{\text{leg}}\right)/\left(\delta^{15}{\text{N}}_{\text{ref}}-B\right)\right]100$$( 2 )

where δ^15^ N_ref_ is the δ^15^ N natural abundance of shoots of the non-N_2_-fixing reference plants (fully dependent on soil N), and δ^15^ Nleg is the δ^15^ N natural abundance of the N_2_-fixing legume. B is the δ^15^ N of shoots of the test legume fully dependent on N_2_-fixation and a measure of isotopic fractionation during N_2_-fixation. The smallest weighted δ^15^ N value for the whole shoot of each legume was used as the B value:–0.80 (cowpea), –1.46 (soybean) and –0.56 (groundnut). The amount of N2 fixed in the whole plant was estimated (using Equation 3) assuming that 30% of the N_2_ fixed was present in the roots (Unkovich *et al*., 2008). The inclusion of N_2_-fixed in below-ground dry matter is vital as it has a strong impact on soil N balance estimation (Peoples *et al*., 2009):

$${\text{Total N}}_{2}-\text{fixed}\left({\text{kg ha}}^{-1}\right)=\left(\%\text{Ndfa }\times \text{ whole shoot N}\right)/0.70.$$( 3 )

$$\text{Net N contributed to the soil N economy }\left({\text{kg ha}}^{-1}\right)\text{was calculated as}$$( 4 )

- Cowpea-maize relay = total N_2_-fixed + applied N − cowpea grain N − maize grain N
- Sole legume = total N_2_-fixed − grain N
- Sole maize = applied N − grain N

### Data analysis

GenStat (version 18.1, VSN International Ltd) was used for statistical analysis. Data for relay and crop rotation systems were initially analysed separately and then combined (for maize data) within a site and across sites for comparisons. Data were analysed with a linear mixed model with cropping sequence, soil fertility and site kept fixed (for cross site analysis) and replication as a random factor. When significant differences between means were observed, the standard error of differences between means (SED) was used to compare treatment means at a significant level of p < 0.05.

## RESULTS

### Soil characteristics and rainfall

Soil organic C, total N, exchangeable K, ECEC and clay content were more favourable for crop growth (p < 0.05) in the SGS than in the NGS (Table 2). Soil organic C and total N were slightly more favourable for crop growth in the HF field than in the LF field, while available P and ECEC were slightly better in the LF field in the SGS. In the NGS, soil C, total N, ECEC and clay content were slightly favourable for crop growth in the HF field relative to the LF field.

**Table 2:** Physical and chemical properties of the three field types used for the trials in the southern Guinea savanna (SGS) and northern Guinea savanna (NGS). See Marinus (2014) for further details.

	SGS				NGS			
Soil fertility characteristic	HF	MF	LF	SED^*^	HF	MF	LF	SED^*^
pH (H_2_O)	5.6	5.7	5.8	0.1	5.2	4.1	5.0	0.5
Organic C (g kg^−1^)	8.2	9.4	6.2	1.3	4.3	3.5	3.9	0.3
Total N (g kg^−1^)	0.8	0.7	0.6	0.1	0.4	0.5	0.3	0.1
Olsen P (mg kg^−1^)	2.2	2.5	3.6	0.6	2.6	4.1	2.7	0.7
Exch. K^+^ (cmol_+_ kg^−1^)	0.2	0.2	0.2	0.01	0.2	0.1	0.2	0.02
Exch. Ca^2+^ (cmol_+_ kg^−1^)	1.4	1.2	1.5	0.1	1.5	0.7	1.0	0.4
Exch. Mg^2+^ (cmol_+_ kg^−1^)	0.6	0.6	0.8	0.1	0.6	0.2	0.6	0.2
ECEC (cmol_+_ kg^−1^)	7.3	6.0	7.9	0.8	4.0	4.0	3.1	0.4
Sand (g kg^−1^)	623	603	503	52	823	863	843	16
Silt (g kg^−1^)	281	301	401	52	121	81	121	19
Clay (g kg^−1^)	96	96	96	0.01	56	56	36	9

* SED represents the standard error of differences between the soil fertility levels, calculated following Saville (2003).

**Figure 2:**
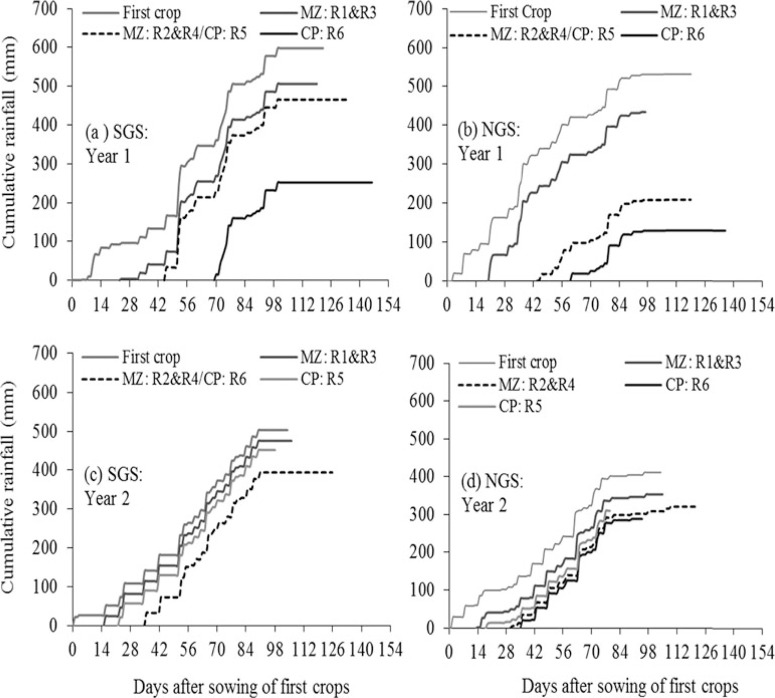
Cumulative rainfall from sowing to harvest time of crops in different cropping sequences in year 1 and year 2 and in the southern Guinea savanna (SGS) and northern Guinea savanna (NGS). In year 1, cowpea in R5 and maize in R2 and R4 were sown on the same day. In year 2, maize in R2 and R4 and cowpea in R6 were sown on the same day in the SGS due to drought at 4 WAS the first crop.

A total of 598 mm rain in year 1 and 503 mm in year 2 were recorded during the growing period in the SGS (Figure 2a and c); 532 mm in year 1 and 423 mm in year 2 were recorded in the NGS (Figure 2b and d). Rainfall was generally well distributed during the growing periods. Relay crops received less rainfall compared with the first crops, with maize in relay R2 and R4 and cowpea in relay R6 (in a descending order) receiving the smallest amount of rainfall in both years.

#### δ^15^N of reference weeds and legumes, shoot N, %Ndfa and N2-fixation

**Figure 3:**
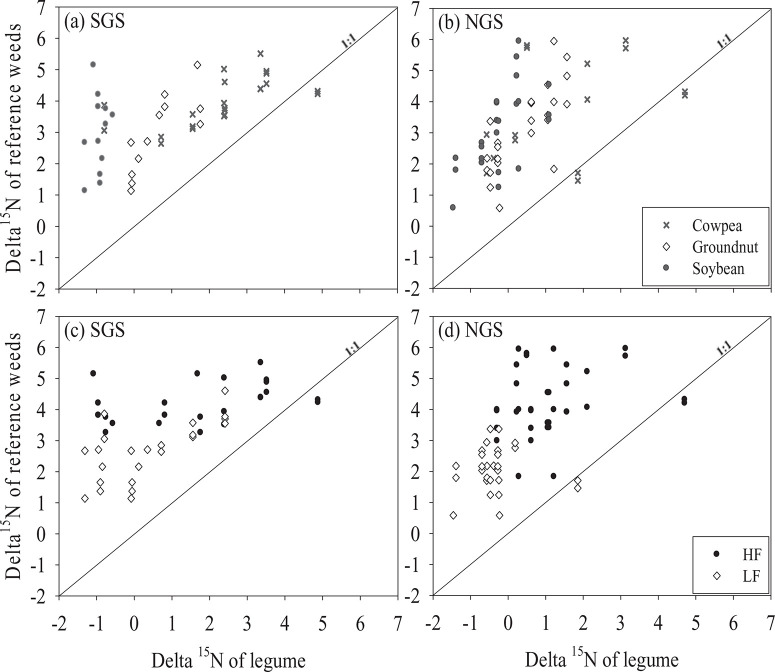
Comparison of δ^15^ N natural abundance (‰) enrichment in grain legumes and in broad-leaved non-N_2_-fixing reference plants as affected by grain legume type (a) and (b), and soil fertility status (c) and (d) in the southern Guinea savanna (SGS) and the northern Guinea savanna (NGS).

The δ^15^ N signatures of the different reference weeds and the legumes did not significantly differ between sites, although the mean values were slightly smaller in the NGS (Figure 3). The reference weeds species had larger δ^15^ N values (p < 0.001) than the legumes (Figure 3a and b). Soybean (−0.9‰) had smaller (p < 0.001) δ^15^ N than cowpea (2.3‰) and groundnut (0.7‰) in the SGS (Figure 3a). In the NGS, the δ^15^ N was comparable between soybean (−0.3‰) and groundnut (0.4‰) but both had smaller (p < 0.01) values than cowpea (1.4‰) (Figure 3b). The δ^15^ N signatures of the reference weeds and legumes were strongly influenced by soil fertility with smaller values (*p <* 0.001) observed in the LF fields (Figure 3c, d).

Above-ground shoot dry matter yields of cowpea and soybean were similar between sites but groundnut accumulated more shoot dry matter (p < 0.001) in the SGS than in the NGS (Table 3). Shoot N content followed a similar trend as shoot dry matter yield with only groundnut producing a 17% larger (p = 0.048) N yield in the SGS. Shoot dry matter yield of soybean was comparable between the HF and LF fields at both sites, whereas cowpea and groundnut produced more shoot dry matter in the HF fields (Table 3).

**Table 3:** Shoot dry matter yield (DM), shoot N, %Ndfa, N uptake, N harvest index (NHI) and soil N balance of different grain legumes and maize (MZ) at different soil fertility levels in the southern Guinea savanna (SGS) and northern Guinea savanna (NGS). Data are for year 1.The SED shows standard error of differences between means for the different crop/cropping systems and soil fertility levels.

Site/fertility	Shoot DM (kg ha^−1^)	Shoot N (kg ^ha−1^)	Ndfa %	N2-fixed/ (kg ^ha−1^)	N applied (kg ^ha−1^)	Grain N (kg ^ha−1^)	Stover N (kg ^ha−1^)	NHI (%)	N balance (kg ^ha−1^)^a^
SGS									
HF field									
CP + MZ (R2)	1541	47	18	12	50	32 + 12	15 + 7	68/63	18
^SB − MZ^	5305	144	88	183	0	142	35	80	41
^GN − MZ^	2361	60	61	53	0	19	16	54	34
^MZ − MZ^				^–^	50	54	17	76	^–4^
Mean	3069	84	56	83	64	23	70	22	
LF field									
CP + MZ (R2)	731	23	58	19	50	14+10	12+6	54/63	45
^SB − MZ^	4676	106	87	131	0	118	26	81	13
^GN − MZ^	2235	55	76	60	0	14	15	49	46
^MZ − MZ^				–	50	34	17	67	16
Mean	2547	61	74	70		48	19	63	30
SED crop	366^***^	11^***^	8^***^	15^***^		9^***^	1^***^	3^***^	n.s.
SED fertility	n.s.	7^*^	7^**^	n.s.		6*	n.s.	2^**^	n.s.
NGS									
HF field									
									
CP + MZ (R2)	1768	56	40	30	25	34 + 0	22 + 0	61/0	21
^SB − MZ^	^5095^	^118^	^67^	^110^	^0^	^137^	^27^	^84^	^−27^
^GN − MZ^	^1896^	^62^	^64^	^57^	^0^	^22^	^17^	^56^	^35^
^MZ − MZ^					^50^	^24^	^8^	^75^	^26^
Mean	2920	79	57	66		54	19	69	14
LF field									
CP + MZ (R2)	1133	29	60	23	25	19+0	17+0	53/0	29
^SB − MZ^	4815	135	86	160	0	66	13	84	94^b^
^GN − MZ^	1173	34	90	43	0	17	13	57	26
^MZ − MZ^					50	7	5	58	43
Mean 2374	66	79	75		27	12	63	48
SED crop	322^***^	11^***^	n.s.	8^***^		6^***^	2^***^	3^***^	n.s
SED fertility			n.s.			5^**^	2^*^	n.s.	9^**^
SED site			n.s.			4^**^	1^***^	n.s.	n.s.

a Soil N balance of cowpea–maize relay (cowpea + maize) in SGS combines the N balances of the cowpea and maize. N balance of cowpea–maize relay in the NGS does not include the N balance of the maize due to failure of maize to produce a grain yield. However, the first N dose of 25 kg ha−1 of urea N was applied to the relay maize in the NGS and added to the soil N balance of the cowpea–maize relay system.

b Soil N balance of soybean in the poorly fertile field in the NGS was possibly an overestimation as destruction by free roaming livestock prior to harvest at maturity led to reduced grain yield and consequently smaller grain N. Shoot DM and shoot N yield of the CP + MZ (R2) are for the CP (cowpea) only.

* Significant at p < 0.05;

** Significant at p < 0.01;

*** Significant at p < 0.001; n.s. = not significant.

Shoot N content, %Ndfa and the total amount of N_2_-fixed by the legumes did not differ between sites (Table 3). Soybean consistently had the largest shoot N content, followed by groundnut and cowpea in a descending order. Shoot N content generally declined with decreasing soil fertility status but the differences were only significant in the SGS (Table 3). The %Ndfa was comparable in soybean and groundnut, while the %Ndfa of cowpea was considerably smaller (p < 0.001) than that of soybean and groundnut at both sites (Table 3). The %Ndfa of legumes grown in the LF fields was greater than in the HF fields (Table 3). The amount of N_2_ fixed was however comparable between LF and HF fields due to a higher biomass production of legumes in the HF fields.

#### Legume and maize N uptake and soil N balance in year 1

Averaged across treatments and soil fertility levels, legume and maize N uptakes in the SGS were 21 kg ha^−1^ and 29 kg ha^−1^ larger than in the NGS (Table 3). The N harvest index (NHI) of the different crops did not differ between sites. Grain N uptake and NHI of groundnut were smaller than those of the other crops (Table 3). Soil fertility affected grain N uptake (p < 0.05, SGS; p < 0.01, NGS) with less N exported through grain in the LF than in the HF fields. As a result, NHI was generally smaller in the LF fields, though the difference was significant only in the SGS (p < 0.01) (Table 3).

The partial soil N balances of legumes were comparable between sites and among the different crops within site (Table 3) though there was a large variability within crops (data not shown). On the other hand, partial N balance of maize was 28 kg ha^−1^ greater (p < 0.001) in the NGS than in SGS. The cowpea–maize relay system had the smallest partial N balance in the NGS, whereas the smallest partial N balance in the SGS was observed in the continuous maize system. The partial soil N balance was larger in the LF fields than in the HF fields at both sites (Table 3). Negative partial N balances were observed only in the HF fields (maize in the SGS and soybean in the NGS).

#### Cropping sequence, maize N uptake and grain yield in year 2

**Figure 4:**
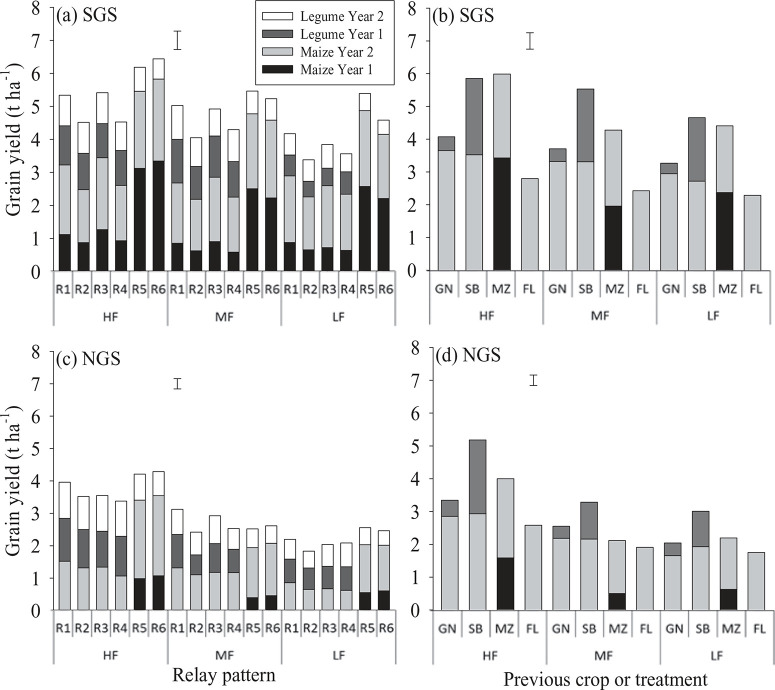
Maize and legume grain yields as influenced by different relay and rotation cropping sequences and soil fertility status in both years in southern Guinea savanna (SGS) and northern Guinea savanna (NGS). Error bars indicate the combined standard error of differences between means for the different cropping patterns across soil fertility status. For an explanation of R1–R6, see Table 1; GN = groundnut; SB = Soybean; MZ = maize; FL = fallow.

Cowpea and groundnut grain yields were comparable between sites (Figure 4). However, the mean grain yield of soybean was 0.69 t ha^−1^ and of maize 0.67 t ha^−1^ larger in the SGS than in the NGS (Figure 4). Spatial arrangement of relay intercrops did not influence legume and maize grain yields (Figure 4a and c). However, relaying cowpea into maize at 3 (R5) and 5 (R6) WAS maize led to a reduction in cowpea grain yield of 28% and 47%, equal to 0.18 and 0.26 t ha^−1^ , in the SGS relative to the mean yield of cowpea in treatments R1–R4 where cowpea was sown as the first crop (Figure 4a). In the NGS, a 29 and 42% reduction in cowpea grain yield, equal to 0.18 and 0.24 t ha^−1^ , relative to the mean yield of cowpea in treatments R1–R4 was observed (Figure 4c). Sowing maize into cowpea at 2 (R1 and R3) and 4 (R2 and R4) WAS cowpea resulted in a 14 and 39% (0.29 and 0.64 t ha^−1^ ) reduction in maize grain yield relative to mean yield of maize in treatments R5 and R6 in the SGS (Figure 4a). The yield penalty was more severe in the NGS where there was a 58 and 83% reduction (0.66 and 0.82 t ha^−1^ ) in maize grain yield sown at 2 and 4 WAS cowpea compared with the mean yield of maize in R5 and R6 (Figure 4c).

Grain yield of maize after natural fallow and in continuous maize did not differ (Figure 4b and d). Maize grain yield increased by 0.89 t ha^−1^ when maize succeeded soybean and by 1.01 t ha^−1^ with groundnut as the preceding crop relative to continuous maize in SGS (Figure 4b). In the NGS, soybean and groundnut increased the grain yield of a subsequent maize by 0.49 and 0.38 t ha^−1^ , respectively (Figure 4d). Cowpea and soybean grain yields were larger in the HF than in the LF fields (Figure 4; p < 0.05 in SGS; p < 0.001 in NGS). Groundnut grain yield was not affected by soil fertility (Figure 4b and d). Soil fertility had no significant impact on maize grain yield in the SGS (Figure 4a and b), while maize yield declined (p < 0.001) with decreasing soil fertility in the NGS (Figure 4c and d).

**Figure 5:**
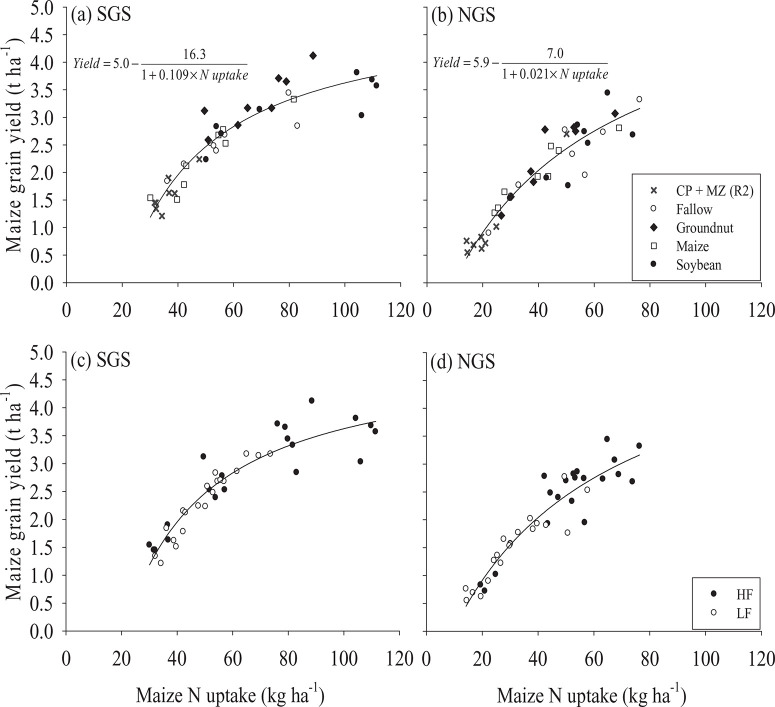
Response of maize grain yield to maize N uptake as influenced by rotation with soybean, groundnut and natural fallow or relay with cowpea or continuous maize in (a) southern Guinea savanna (SGS), (b) northern Guinea savanna (NGS), and under different soil fertility status in the (c) SGS and the (d) NGS of northern Ghana. CP + MZ (R2) refers to the cowpea–maize relay treatment with maize sown 4 WAS cowpea.

N uptake by maize was greater in the SGS than in the NGS (Figure 5). Grain yield of maize had a curvilinear relationship with maize N uptake and the yield seemed to reach a plateau at 80–120 kg ha^−1^ of N uptake, particularly in the SGS (Figure 5a and c). The fitted model explained 87% (SGS) and 88% (NGS) of the variability in maize grain yield. The model suggested a maximum grain yield of 5.0 t ha^−1^ in the SGS and 5.9 t ha^−1^ in the NGS that can be achieved when N is not limiting on farmers’ fields (Figure 5a and b). Soybean and groundnut increased (p < 0.001) N uptake by subsequent maize compared with continuous maize in the SGS (Figure 5a). In the NGS, only soybean stimulated a 33% larger uptake of N (p < 0.001) by the succeeding maize relative to continuous maize (Figure 5b). Mean N uptake by maize following natural fallow was comparable with that of continuous maize at both sites. Relay maize sown at 4 WAS cowpea decreased maize N uptake by 36% in the SGS and 48% in the NGS compared with continuous maize (Figure 5a and b). Crops in HF fields had a greater maize N uptake (p < 0.05 in SGS; p < 0.01 in NGS) than in LF fields (Figure 5c and d).

#### Cumulative crop yields

Crops in the SGS provided more stover and grain yield than in the NGS over the two years (Figures 3 and 5). The cumulative grain yield was comparable between relay treatments R5–R6 and soybean–maize rotation, while these treatments provided larger grain yields than the other cropping sequences (Figure 4). The crops grown in the HF fields consistently yielded more maize and legume grain in rotation and in relay compared with the MF and LF fields at both sites (Figure 4).

**Figure 6:**
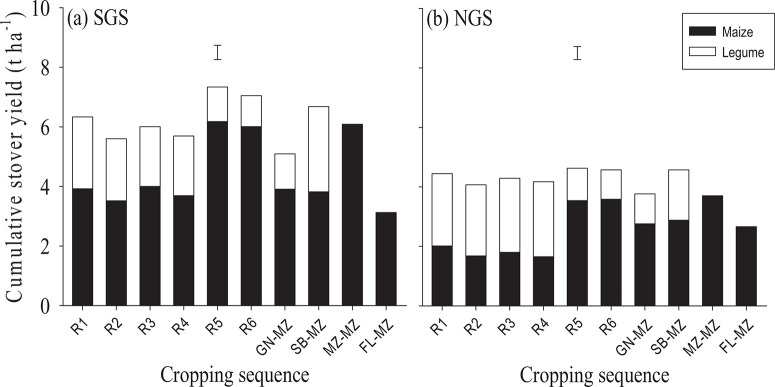
Accumulated maize and legume stover yields over both years averaged across soil fertility for the different cropping sequences in southern Guinea savanna (SGS) and northern Guinea savanna (NGS). Error bars indicate the combined standard error of differences between means.

Cumulative stover yield followed a similar pattern as grain yield with more stover in the SGS than the NGS (Figure 6), and declines in stover yield with decreasing soil fertility at both sites (data not shown). Soybean–maize rotation and relay R5–R6 provided larger cumulative stover yields than the other cropping sequences (Figure 6).

### DISCUSSION

#### N2-fixation and net N contribution to the soil for a subsequent maize crop

The greater δ^15^ N values of the reference weed species compared with the legumes indicates %Ndfa by the legumes were estimated reliably using the natural abundance method (Unkovich *et al*., 2008). Differences in the application of crop residues and mineral fertilisers and their effects on N turnover possibly resulted in a larger soil organic C and total N in the HF fields compared with the LF fields (Table 2). This could account for the larger δ^15^ N enrichment of the reference weeds and the legumes in the HF fields compared with the LF fields (Figure 3c and d; Unkovich *et al*., 2008).

The observed %Ndfa values (Table 3) are consistent with findings by others (e.g. Giller, 2001; Kermah *et al*., 2018) that poor soil fertility stimulates grain legumes to rely more on atmospheric N_2_-fixation for growth. The amount of N_2_ fixed by a legume is related to the %Ndfa as well as shoot biomass and N yield (Giller, 2001; Kermah *et al*., 2018; Peoples *et al*., 2009). The large shoot biomass and N yields in the HF fields explain why comparable amounts of N_2_ were fixed in the HF and the LF fields, despite a greater %Ndfa of legumes in LF fields (Table 3).

The negative soil N balances of cowpea in HF fields at both sites and of soybean in the HF field in the NGS reflected their %Ndfa being smaller than the NHI (Table 3). A maize crop succeeding a grain legume only benefits from a fixed-N effect if the preceding grain legume relies more on atmospheric N_2_-fixation than on soil N for its growth (Giller, 2001). Nevertheless, N sparing by a grain legume could also result in enhanced N availability to a succeeding crop or a crop grown in association with a grain legume, despite a negative soil N balance of the grain legume. This explains the increased maize grain yield after soybean in the HF field in the NGS, similar to thatof maize following groundnut with a positive N balance. Such increased grain yield of maize after soybean could also be due to non-N benefits (e.g. increased soil microbial biomass and functioning, improved soil structure, improved N mineralisation) (Franke *et al*., 2018; Giller, 2001, Sanginga *et al*., 2002; Yusuf *et al*., 2009b). Residual effects of fixed N_2_ on maize performance are likely to be more important in LF fields than in HF fields, but the results from the current trial do not provide clear evidence for this.

#### Performance of relay crops as affected by biophysical properties

The greater productivity of the crops grown in the SGS and in the HF fields were consistent with their favourable soil fertility properties (Table 2; Figures 4 and 6). The LF field in the SGS was marked by poor drainage that may have led to denitrification, reduced nutrient availability and uptake by the crops. Maize in the MF field in the SGS was affected by Striga hermonthica, which affected grain yield particularly in year 1 (Figure 4a and b). The MF field in the NGS was also characterised by poor drainage, which may have restricted nutrient availability and uptake by the crops. Soil organic C, total N and available P appeared to be sub-optimal for crop growth in all fields (Fairhurst, 2012). However, the deficiencies of total soil N and available P were most probably corrected by P application in both years, N application in year 1 and residual N benefits in year 2 (Table 3).

The present results show that the time of relay sowing of maize and cowpea had an overriding influence on grain yield, as grain yield declined consistently with a delay in sowing of the relay crops regardless of the within-row spatial arrangement of the crops (Figure 4a and c). This overriding effect, in the case of maize could mainly be due to the decreasing amount of rainfall received by the relay crops for growth (Figure 2; Table 1). In the case of the cowpea relayed into maize, shading of the cowpea by maize likely contributed to the observed cowpea grain yield reduction particularly in the SGS where the maize produced larger biomass evidenced by the greater maize stover yield (Figure 6a). In this case, the first maize crop could be sown early in the season (with early onset of rainfall), and the relay cowpea sown when the maize leaves begin to senesce. This could reduce the shading of the relay cowpea by the maize, which could improve the productivity of the cowpea and the overall relay system. Also, the cowpea in R1–R4 can be sown early in the season so that the relay maize could receive enough rainfall during the growing season to mature, which could improve the yield and the productivity of that relay system. In such instances, the relay cowpea could be harvested early in the season to provide food for the farm households while awaiting the main harvest later in the season.

However, early sowing of cowpea could result in flowering, pod setting and maturation coinciding with the peak of rainfall in August leading to high diseases and pests pressure reducing the yield, in addition to poor drying of pods. The ideal sowing times of the relay crops is also likely to vary from season to season and between sites in the Guinea savanna depending on the onset of the rainy season. This could affect the relative productivity of the different cropping sequences, particularly the relay cropping patterns. Therefore, a modelling work based on our results is needed to explore and possibly identify ideal sowing times and associated risks for the different sites in the Guinea savanna.

The failure of the relay maize sown at 3 and 6 WAS cowpea in the NGS in year 1 was mainly due to the poor quality of the seeds used for sowing compounded by insufficient rainfall. Insufficient rainfall largely accounted for the failure of the relay cowpea sown at 6 and 9 WAS the maize in year 1 in the NGS as only 209 mm (R5) and 130 mm (R6) rainfall were received (Figure 2b). In the SGS, the relay cowpea failed in year 1 due to severe infestation by bacteria blight disease (*Xanthomonas axonopodis pv. vignicola (Xav*). First, the infestation presumably resulted from infected seeds, and second, infections after germination proliferated due to the consistent rainfall during the early stages of growth (Figure 2a; de Lima-Primo *et al*., 2015). These show the difficulty in identifying the optimal sowing time for cowpea in the Guinea savanna as the crop could be affected by too little or too much rain, which can possibly lead to crop failure. The broad range of causes of crop failure also highlights the usefulness of crop diversification in smallholder farming systems in the Guinea savanna.

#### Effect of cropping sequence on crop productivity

The apparent plateauing of maize grain yield at 80–120 kg ha^−1^ of N uptake in the SGS (Figure 5a and c) suggests that increased supply of N beyond that rate may not lead to appreciable maize grain yield increases. Marginal increases and subsequent plateauing of maize grain yield after N input of 30–40 kg ha^−1^ on a highly infertile soil have been reported in the Guinea savanna of Nigeria (Franke *et al*., 2004) and in East Africa (Ndungu-Magiroi *et al*., 2017). N seemed to be less limiting for maize yield in the groundnut/soybean–maize rotation than in the continuous maize system indicated by the 9–13 kg ha^−1^ greater N uptake in maize that succeeded groundnut and soybean (Table 3).

The comparable yield of maize after natural fallow and continuous maize has previously been reported in the Guinea savanna of Nigeria (Franke *et al*., 2008; Yusuf *et al*., 2009a, 2009b). This implies that a one-year fallow period in the Guinea savanna is not suitable for soil fertility regeneration to increase and sustain crop yields as a one year fallow is too short to restore soil fertility when the growing season is short (Franke *et al*., 2008; Yusuf *et al*., 2009b). The similar grain yield of maize succeeding soybean or groundnut suggests that the choice of a grain legume species as a preceding crop was not an important factor in this study, as also observed by Sauerborn *et al*. (2000) in the Guinea savanna of northern Ghana. The better maize grain yield in year 2 in the NGS (Figure 4c and d) was largely the outcome of better quality seeds used for sowing in year 2. The decline in grain yield of continuous maize in year 2 relative to year 1 in the SGS could be attributed to the negative (HF field) or smaller (LF field) soil N balance in year 1 (Table 3).

The superior maize grain yield induced by soybean and groundnut as preceding crops compared with continuous maize and the other cropping sequences (Figure 4b and d) stresses why legume-maize rotation predominates smallholder farming systems in the Guinea savanna. However, the present results indicate that sowing maize first and relaying cowpea into it (R5 and R6; despite the relay cowpea failing to produce yield in year 1) represents an alternative to the legume-maize rotations for smallholder farms in the Guinea savanna. Nonetheless, the soybean–maize rotation provided more legume grain than the relay systems (Figure 4) which is vital for household nutrition and income as legume grain has higher nutritional and economic value than maize (Giller, 2001; Kermah *et al*., 2017). The poor yield of groundnut (Figures 4b, d and 6) was the outcome of late sowing due to late onset of rainfall. This led to the smaller total productivity of the groundnut–maize rotation relative to the soybean–maize rotation and the R5 and R6 relay systems.

### CONCLUSIONS

Low soil fertility stimulates grain legumes to rely more on atmospheric N_2_-fixation than on soil N for growth resulting in larger partial soil N balances of grain legumes grown in the LF fields. A rotation of soybean or groundnut with maize is superior in increasing subsequent maize yield than natural fallow rotation and relay cropping of maize and cowpea. Relaying cowpea into maize is more productive than relaying maize into cowpea. The productivity of relay cropping where maize is sown first and cowpea planted much later seems a promising ecological intensification option alternative to the dominant legume-maize rotation in the Guinea savanna. Relaying cowpea into maize is thus recommended in the Guinea savanna when the growing season is short due to late onset of rainfall as was observed during this study.
